# The Pandemic of COVID-19: Current Situation in South Africa

**DOI:** 10.1017/dmp.2021.61

**Published:** 2021-03-04

**Authors:** Shahrokh Hatefi, Farouk Smith, Khaled Abou-El-Hossein, Javad Alizargar

**Affiliations:** 1 Precision Engineering Laboratory, Department of Mechatronics, Nelson Mandela University, Port Elizabeth, South Africa; 2 Research Center for Healthcare Industry Innovation, National Taipei University of Nursing and Health, Sciences, Taipei City, Taiwan

**Keywords:** COVID-19, SARS-COV-2, South Africa, public health

Since March 11, 2020, the pandemic of a novel coronavirus (now named severe acute respiratory syndrome coronavirus 2 [SARS-CoV-2], the cause of the disease COVID-19) was announced by the World Health Organization (WHO).^1^ According to the WHO coronavirus disease live dashboard, there have been 104,370,550 confirmed cases of COVID-19, including 2,271,180 deaths, as of 5:00 pm CEST, February 5, 2021 (https://covid19.who.int). In almost all countries, COVID-19 and associated problems have severely affected global health, especially in middle- and low-income countries with limited resources, including the Middle-East and African countries.^2^ Countries across Africa with previous pandemic disease outbreaks, where contagious diseases, such as Ebola and HIV, are continuing to spread, are still exhibiting weak public health-care systems, low medical resources, bad health management systems, and limited financial means. It has been reported that direct and indirect influences of highly contagious diseases, including COVID-19, have severely affected the maternal and infant cases in Africa.^3^

During the current pandemic, the first positive case in Africa was confirmed in Egypt, on February 14, 2020. With regard to the limited facilities, as well as high prevalence of other disease and viruses, including HIV, malaria, and tuberculosis in Africa, collective and prompt responses for allocating limited resources to fight COVID-19, and controlling the spread rate of viruses are crucial. According to the International Health Regulation (IHR) monitoring and evaluation framework, South Africa, Egypt, and Algeria have the highest risk in terms of importation rate, and have an average risk profile in responding to highly contagious disease outbreaks.^4^ In South Africa, the government announced a national disaster on March 15, 2020. Since March 26, South Africa entered a nationwide lockdown. Social distancing rules, handwashing, sanitizing, monitoring, diagnosing, and tracking the connection record of COVID-19 patients, as well as banning the sale of alcohol and tobacco products were some of the important decisions that were made by the government in South Africa. Different monitoring strategies, including airport and shopping center monitoring, have been performed to detect infected individuals and preventing the spread of this deadly virus.

South Africa is home to approximately 59 million people, 66.7% of whom live in urban areas (39,550,889 people).^5^ The health-care system there has shown an average case finding rate (positive case/tests conducted) of 17.43%; with 8,436,569 cumulative tests and a case fatality rate of 3.12%.^6^ South Africa is approximately 12.4% the size of the United States of America; however, the lack of local laboratories and health-care centers for diagnosing and treating people is significantly affecting the health-care quality. There is a delay between sampling and the outcome of the COVID-19 test in many locations in South Africa, which negatively affects the treatment, isolating, and follow-up strategies. In addition, implementing the lockdown strategy as well as COVID-19 measures have impacted the HIV care continuum.^7^ Another concern is the shortage of skilled health-care workers, especially in the urban areas and other poor locations, as the current pandemic requires skilled health-care workers and essential health facilities.

South Africa is still endemic for disease outbreaks, with 1,483,345 confirmed COVID-19 cases, 45,902 deaths, 93,976 active cases, and 1,327,186 recoveries as of the February 6, 2021.^6^ There have been more than 3751 new cases and 261 deaths reported in the past 24 h in South Africa, on February 6, 2021.^6^ In addition, there are other concerns in the current situation of South Africa; around 45,000 children under the age of 10 are already COVID-19 positive; in some regions, the process of the COVID-19 test takes up to 10 days.

Low levels of safety as well as high financial insecurity in many poor locations and urban areas have severely affected the provision and coverage of public health care. Therefore, educating the community about hygiene practices, and improving the public and healthcare staff knowledge, as well as using novel techniques based on advanced technologies for diagnosing, monitoring, decision-making, and management purposes are urgently required.

Since the WHO announced the pandemic of SARS-CoV-2/COVID-19, individuals traveled back to South Africa from European countries and China, with a high risk of having been infected by SARS-CoV-2. However, polymerase chain reaction (PCR) tests were taken from people who have returned to South Africa. The first known COVID-19 confirmed case was a South African returning from Italy. As a result, the authorities have locked down the country, banned travelling, and restricted almost all social and economic activities. However, like many other countries, South Africa has been facing problems with continuing the lockdown strategy.^8^

The lockdown has been eased twice since the announcement of a national disaster and the commencement of the national lockdown; however, it has been aggravated again by the increasing number of positive cases. Furthermore, with regard to the previous pandemic of HIV in Africa, it could be deduced that such contagious viruses would carry on affecting poorer people, children, and young women severely.^3,7^ Therefore, we strongly urge the unity of the international community and regional associations to have collaborative efforts in fighting the COVID-19 pandemic by implementing strategies and management solutions to decrease the spread rate of the virus in South Africa, Sub-Saharan, African countries, and the greater global community.
